# Supplementary material for: Polyketide synthase gene clusters in the ericoid mycorrhizal fungus Oidiodendron maius: genome-wide prediction, expression profiling, and functional characterization of knockout mutants
Source: Front Plant Sci. 2026 Sep 16;17:1924430. doi: 10.3389/fpls.2026.1924430 (PMC13623903; doi:10.3389/fpls.2026.1924430)
Supplement: Supplementary file 1 [file SupplementaryFile1.zip › Supplementary Materials/Supplementary Folder 1.zip!/Oidma1_AssemblyScaffolds_Repeatmasked/knownclusterblast/cluster53/jgi.p_Oidma1_138176_mibig_hits.html]

| MIBiG Protein | Description | MIBiG Cluster | MiBiG Product | % ID | % Coverage | BLAST Score | E-value |
| --- | --- | --- | --- | --- | --- | --- | --- |
| OAQ83753.1 | sterigmatocystin\_8-O-methyltransferase | BGC0001358 | Leucinostatins\_biosynthetic\_gene\_cluster | 33 | 98.1981981982 | 228 | 1.5e-59 |
| ADM79461.1 | O-methyltransferase | BGC0001266 | Grayanic\_acid\_biosynthetic\_gene\_cluster | 31 | 97.7477477477 | 203 | 3.9e-52 |
| CCH26291.1 | putative\_O-methyltransferase | BGC0001158\_c2 | Bikaverin\_biosynthetic\_gene\_cluster | 29 | 99.3243243243 | 183 | 7.1e-46 |
| CCT67993.1 | bikaverin\_cluster-O-methyltransferase | BGC0000030 | Bikaverin\_biosynthetic\_gene\_cluster | 28 | 99.7747747748 | 181 | 2.1e-45 |
| ABK64180.1 | O-methyltransferase | BGC0001247\_c3 | Cercosporin\_biosynthetic\_gene\_cluster | 31 | 100.900900901 | 176 | 6.6e-44 |
